# Effect of Calcium-Sulphate-Bicarbonate Water in a Murine Model of Non-Alcoholic Fatty Liver Disease: A Histopathology Study

**DOI:** 10.3390/ijms231710065

**Published:** 2022-09-02

**Authors:** Guido Carpino, Diletta Overi, Paolo Onori, Antonio Franchitto, Vincenzo Cardinale, Domenico Alvaro, Eugenio Gaudio

**Affiliations:** 1 Department of Movement, Human and Health Sciences, University of Rome “Foro Italico”, 00135 Rome, Italy; 2 Department of Anatomical, Histological, Forensic Medicine and Orthopedic Sciences, Sapienza University of Rome, 00161 Rome, Italy; 3 Department of Medico-Surgical Sciences and Biotechnologies, Sapienza University of Rome, 04100 Latina, Italy; 4 Department of Translational and Precision Medicine, Sapienza University of Rome, 00185 Rome, Italy

**Keywords:** natural mineral water, liver disease, lipotoxicity, lipopolysaccharide, steatosis, inflammation

## Abstract

The progression of nonalcoholic fatty liver disease (NAFLD) is associated with alterations of the gut–liver axis. The activation of toll-like receptor 4 (TLR4) pathways by endotoxins, such as lipopolysaccharide (LPS), contributes to liver injury. The aim of the present study was to evaluate the possible beneficial effects of a calcium-sulphate-bicarbonate natural mineral water on the gut–liver axis by evaluating liver and terminal ileum histopathology in a murine model of NAFLD. NAFLD was induced in mice by administrating a methionine-choline-deficient (MCD) diet. The following experimental groups were evaluated: controls (N = 10); MCD+Tap water (MCD; N = 10); MCD+Calcium-sulphate-bicarbonate water (MCD/W_csb_; N = 10). Mice were euthanised after 4 and 8 weeks. Liver and terminal ileum samples were collected. Samples were studied by histomorphology, immunohistochemistry, and immunofluorescence. In mice subjected to the MCD diet, treatment with mineral water improved inflammation and fibrosis, and was associated with a reduced number of activated hepatic stellate cells when compared to MCD mice not treated with mineral water. Moreover, MCD/W_csb_ mice showed lower liver LPS localization and less activation of TLR4 pathways compared to the MCD. Finally, W_csb_ treatment was associated with improved histopathology and higher occludin positivity in intestinal mucosa. In conclusion, calcium-sulphate-bicarbonate water may exert modulatory activity on the gut–liver axis in MCD mice, suggesting potential beneficial effects on NAFLD.

## 1. Introduction

Nonalcoholic fatty liver disease (NAFLD), or metabolic-associated fatty liver disease (MAFLD) is a chronic liver disease defined by the presence of lipid accumulation within hepatocytes without excessive alcohol consumption [1,2]. NAFLD represents the most common cause of liver disease, affecting ~25% of the population, with an increasing global prevalence [3,4]. NAFLD development results from a variety of metabolic, genetic, and environmental factors, which ultimately lead to fat overload in the liver and an inadequate handling of metabolic substrates by the hepatocytes [2]. In particular, liver steatosis drives hepatocellular injury due to an altered cellular energetic metabolism, abnormal lipid compounds, and reactive oxygen species, in a process that is overall defined as “lipotoxicity” [5]. In this context, inflammation and regenerative pathways occurring within the liver are at the basis of the progression from a fatty liver towards nonalcoholic steatohepatitis (NASH), which is characterized by the development of severe inflammation and, eventually, fibrosis progressing towards cirrhosis [6,7].

Recent evidence has demonstrated the relevance of the gut–liver axis in NAFLD pathogenesis [8]. It has been shown that NAFLD is strictly associated with alterations in gut microbiota and intestinal permeability [9]. This can, in turn, affect liver inflammation via the translocation of damage- and pathogen-associated molecular patterns (DAMPs and PAMPs, respectively) and, particularly of bacterial compounds (i.e., endotoxins), to the liver through portal circulation [10]. In this light, *Escherichia Coli*-derived lipopolysaccharide (LPS) has been identified as a driver of liver inflammation both in experimental models and in human NAFLD [11]; its deleterious effects are largely mediated by the activation of toll-like receptor 4 (TLR4) pathways within the liver [10]. Therefore, the modulation of gut physiology and microbiota composition in NAFLD patients might represent a therapeutic approach to attenuate liver inflammation and, eventually, slow disease progression towards NASH. Currently, several pharmacological strategies, targeting specific pathogenetic mechanisms of NAFLD, have been tested or are under study [4,12]; however, no definite therapeutic approach has been validated yet, and lifestyle intervention remains the cornerstone of NAFLD management, at least for dysmetabolic and/or obese patients [12,13].

Natural mineral waters represent a valuable means for the dietary intake of essential elements. Compared to tap water or purified water [14,15], natural mineral waters are highly enriched in minerals and elements, such as calcium, carbonic metabolites, sodium chlorite, sulphates, and/or iron, in variable proportions according to the water source [16]. Interestingly, hydropinotherapy (i.e., administration of natural mineral water for therapeutic purposes) has been proposed as a useful support in the management of several gastrointestinal and hepatobiliary conditions, based on the observed effects in the modulation of lipid profiles and metabolisms; intestinal inflammation; oxidative stress; and bile acid composition [17,18,19]. In this setting, the positive effects of natural mineral water administration on histopathological features of NAFLD are yet to be investigated.

Therefore, the aim of the present study was to evaluate the effects of calcium-sulphate-bicarbonate water (W_csb_) in a mouse model of NAFLD (i.e., methionine-choline-deficient diet–MCD) and, in particular, to assess: (i) eventual improvements to liver histopathology (i.e., liver steatosis, inflammation and fibrosis); (ii) modification of LPS liver localization; (iii) TLR4 pathway activation; and iv) improvements of terminal ileum histopathology and modification of occludin positivity in enterocytes.

## 2. Results

### 2.1. Histopathology of MCD Mice

**Figure 1:**
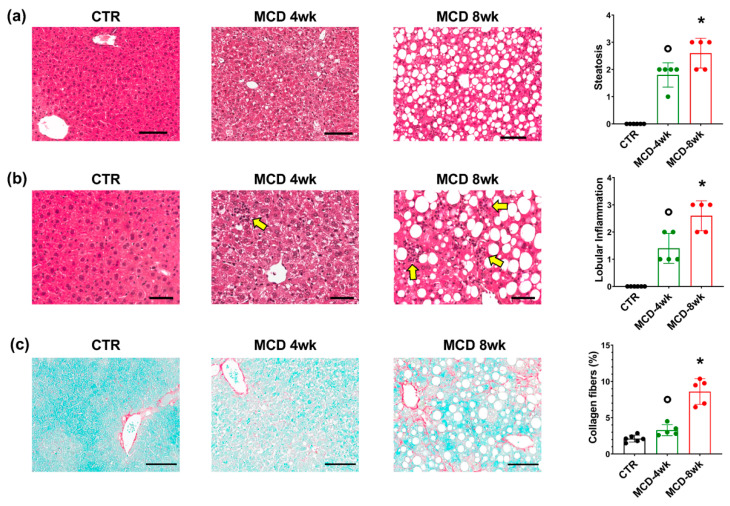
Histopathology of mice after methionine-choline-deficient (MCD) diet administration. (**a**,**b**) Hematoxylin and eosin (H&E) stain on MCD and control (CTR) mouse livers. In MCD mice, steatosis (**a**) and lobular inflammation ((**b**), arrows) progressively increases after four weeks (4wk) and eight weeks (8wk) of MCD diet administration. I. Sirius red/fast green (SR/FG) stains in MCD and CTR mouse livers. Collagen deposition in mice livers increased after eight weeks of MCD diet administration. Scale bars: 100 μm (**a,c**) and 50 μm (**b**). In (**a**–**c**), histograms show means and standard deviations for steatosis and inflammation scores (**a**,**b**), and for the area percentage of the liver occupied by collagen fibers (**c**). Data from each animal are plotted as solid circles in the graph. * *p* < 0.05 vs. all other groups; ° *p* < 0.05 vs. CTR.

The microscopic examinations (Figure 1) showed that MCD mice that underwent 4 weeks of treatment (MCD-4wk) were characterized by the appearance of significant steatosis (score = 1.6 ± 0.6), lobular inflammation (score= 1.4 ± 0.6), and fibrosis (score = 1.4 ± 0.6) compared to controls (score = 0; *p* < 0.001).

Moreover, MCD mice were characterized by higher collagen deposition within liver parenchyma (2.9 ± 1.1%) compared to controls (2.1 ± 0.5%; *p* = 0.013). Compared to MCD-4wk, MCD mice that underwent 8 weeks of treatment (MCD-8wk) showed significantly higher steatosis (score = 2.6 ± 0.6; *p* = 0.020), lobular inflammation (score = 2.6 ± 0.6; *p* = 0.008) and fibrosis (score = 2.6 ± 1.77; *p* = 0.008). Furthermore, MCD-8wk mice were characterized by significantly higher collagen deposition (8.6 ± 1.8%) compared to MCD-4wk (*p* < 0.001).

### 2.2. Effects of Calcium-Sulphate-Bicarbonate Water (W_csb_) on Liver Histopathology in MCD Mice

**Figure 2:**
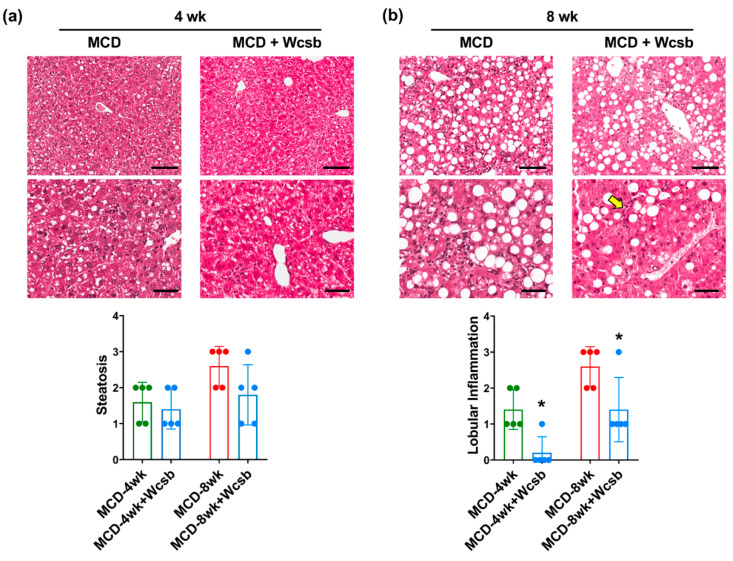
Histopathological changes in mice subjected to a methionine-choline-deficient (MCD) diet and treated with calcium-sulphate-bicarbonate water (W_csb_). (**a**,**b**)**.** Hematoxylin and eosin (H&E) stain on mouse livers after four weeks (4wk, (**a**)) or eight weeks (8wk, (**b**)) of MCD diet administration with or without W_csb_. In MCD mice, no significant difference was observed in terms of liver steatosis (**upper panels**) after W_csb_ administration. Lobular inflammation (lower panels, arrows) was reduced after W_csb_ administration compared to untreated MCD mice. Scale bars: 100 μm (**upper panels**) and 50 μm (**lower panels**). Histograms show means and standard deviations for steatosis and inflammation scores. Data from each animal are plotted as solid circles in the graph. * *p* < 0.05 vs. MCD.

**Figure 3:**
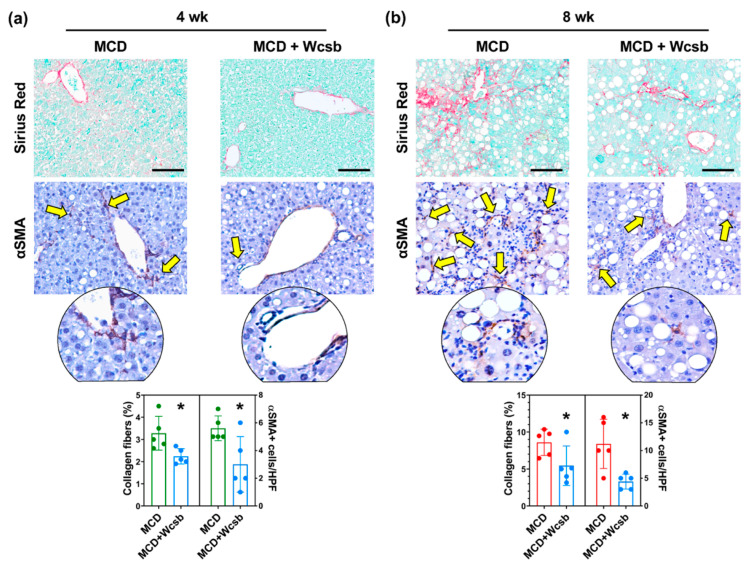
Effects of calcium-sulphate-bicarbonate water (W_csb_) on fibrosis and hepatic stellate cell (HSC) activation in mice subjected to a methionine-choline-deficient (MCD) diet. (**a**,**b**) Sirius red/fast green (SR/FG) stain (upper panels) and immunohistochemistry for α-smooth muscle actin (αSMA) on mouse livers after four weeks (4wk) or eight weeks (8wk) of MCD diet administration, with or without W_csb_. Mineral water administration was associated with lower collagen fiber deposition and with a lower number of activated αSMA+ HSCs (arrows) in MCD mice. Scale bars: 100 μm (**upper panels**) and original magnification: 20× (**lower panel**). Areas in the circles are 40× magnifications. Histograms show means and standard deviations for the area percentage of the liver occupied by collagen fibers and for the number of αSMA+ HSCs per high-powered field (HPF). Data from each animal are plotted as solid circles in the graph. * *p* < 0.05 vs. MCD.

All MCD mice drinking calcium-sulphate-bicarbonate water (MCD/W_csb_) were characterized by a certain degree of NASH histopathological features (i.e., steatosis, lobular inflammation, and fibrosis; Figure 2 and Figure 3). Among mice sacrificed after 4 weeks of treatment, MCD/W_csb_ mice showed significantly lower inflammation (score= 0.25 ± 0.5), fibrosis (score= 0.25 ± 0.5) and collagen deposition (2.3 ± 0.4%) compared to MCD-4wk mice (*p* < 0.05); no significant differences were observed in terms of steatosis (score= 1.3 ± 0.5) compared to MCD-4wk mice.

When the mice that had been euthanised after 8 weeks of treatment were analysed, MCD/W_csb_ mice showed significantly less inflammation (score= 1.4 ± 0.9) compared to MCD-8wk mice (*p* = 0.034). No statistically significant differences were observed in terms of steatosis (score= 1.8 ± 0.8), fibrosis (score= 2.6 ± 0.5), and collagen deposition (5.4 ± 2.7%); however, 3 out of 5 MCD/W_csb_ mice were characterized by lower steatosis and fibrosis scores compared to the MCD group.

### 2.3. Effects of W_csb_ on Fibrogenetic Cells in MCD Mice Liver

The activation of hepatic stellate cells (HSCs) was studied using immunohistochemistry for alpha-smooth muscle actin (αSMA). Among the mice euthanised after 4 weeks of treatment (Figure 3), MCD-4wk mice showed an increase in the number of αSMA+ HSCs (5.8 ± 0.9) compared to the control mice (1.0 ± 0.7; *p* < 0.001); moreover, MCD/W_csb_ mice showed a lower number of αSMA+ cells (3.0 ± 2.0) compared to MCD-4wk mice (*p* = 0.041).

When the mice euthanised after 8 weeks of treatment were analysed, MCD-8wk mice showed a significantly higher number of αSMA+ HSCs (11.2 ± 4.4) compared to MCD-4wk mice (*p* = 0.048) and controls (*p* < 0.001). At 8wks, MCD/W_csb_ mice showed a significantly lower number of αSMA+ HSCs (4.4 ± 1.3) compared to MCD-8wk mice (*p* = 0.011).

### 2.4. Hepatocyte LPS Localization and TLR4+ Macrophages in the Liver of MCD Mice

**Figure 4:**
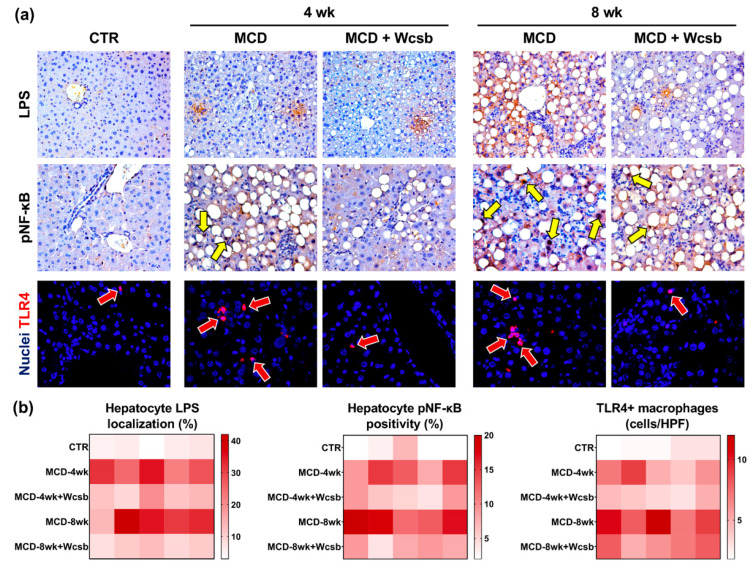
Lipopolysaccharide (LPS) hepatocyte localization and toll-like receptor (TLR)4 pathways in the livers of mice subjected to a methionine-choline-deficient (MCD) diet and treated with calcium-sulphate-bicarbonate water (W_csb_). (**a**) Immunohistochemistry for LPS (upper panels), phospho-nuclear factor-κB (pNF-κB) (middle panels), and immunofluorescence for TLR4 (lower panels) in controls (CTR), and in MCD mouse livers, after four weeks (4wk) or eight weeks (8wk) of MCD diet administration, with or without W_csb_. W_csb_ administration in MCD mice was associated with lower hepatocyte LPS localization, nuclear pNF-κB positivity (yellow arrows) and a reduced number of infiltrating TLR4+ macrophages (red arrows). Original magnification: 10× (immunohistochemistry) and 20× (immunofluorescence). In immunofluorescence, nuclei are in blue. (**b**) Heat maps report data from all animals for the percentage of LPS+ and pNF-κB+ hepatocytes, and for the number of TLR4+ macrophages. Each cell represents an individual animal.

LPS localization in hepatocytes was studied using immunohistochemistry stains of liver sections (Figure 4). Hepatocyte LPS localization was higher in MCD-4wk mice (28.4 ± 5.2%) and in MCD-8wk mice (31.0 ± 10.4%) compared to controls (6.0 ± 2.6%; *p* < 0.001); no differences were present among MCD-4wk and MCD-8wk mice. When mice sacrificed after four weeks of treatment were analysed, MCD/W_csb_ mice showed a lower LPS hepatocyte content (13.6 ± 3.9%) compared to MCD-4wk mice (*p* < 0.001). Similarly, at eight weeks, MCD/W_csb_ mice showed a significantly lower number of LPS+ hepatocytes (10.4 ± 2.0%) compared to MCD-8wk mice (*p* = 0.002).

When the phosphorylated (i.e., active) form of NF-κB was studied, we observed that hepatocytes in MCD mice were characterized by a higher nuclear positivity for pNF-κB in MCD-4wk mice (12 ± 3.3) and MCD-8wk mice (16 ± 3.4) compared to controls (3.2 ± 2.2; *p* = 0.001 and *p* < 0.001, respectively); no differences were present between MCD-4wk and MCD-8wk mice. Nuclear positivity for pNF-κB by hepatocytes was lower after in MCD/W_csb_ (6.8 ± 2.6) compared to MCD mice (*p* = 0.025) after four weeks. At eight weeks, MCD/W_csb_ mice showed a significantly lower number of pNF-κB+ hepatocytes (7.6 ± 2.1) compared to MCD-8wk mice (*p* = 0.001).

The number of TLR4+ macrophages (Figure 4) was increased in MCD-4wk mice (6.2 ± 1.9) and MCD-8wk mice (9.3 ± 2.0) compared to controls (2.7 ± 0.6; *p* < 0.01); the difference in TLR4+ macrophages in MCD-8wk mice and MCD-4wk mice was statistically significant. When mice euthanised after 4 weeks of treatment were analysed, MCD/W_csb_ mice showed a lower TLR4+ macrophage number (4.0 ± 0.8) compared to MCD-4wk mice (*p* = 0.044). Similarly, at 8 weeks, MCD/W_csb_ mice showed a significantly lower number of TLR4+ macrophages (6.8 ± 1.3) compared to MCD-8wk mice (*p* = 0.047).

### 2.5. Effects of W_csb_ on the Intestine in MCD Mice

**Figure 5:**
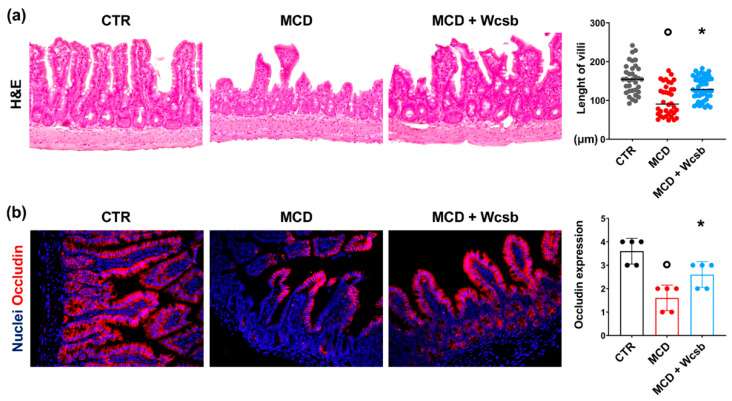
Histopathological and phenotypical modifications in the terminal ileum of mice subjected to a methionine-choline-deficient (MCD) diet and treated with calcium-sulphate-bicarbonate water (W_csb_). (**a**) Hematoxylin and eosin (H&E) stain on the ileum of mice after 8 weeks of MCD diet, with or without W_csb_ administration, and in controls (CTR). Villi were shorter in MCD mice compared to CTR; however, W_csb_ administration was associated with longer villi compared to untreated MCD mice. Original magnification: 10×. (**b**) Immunofluorescence for occludin on the ileum of MCD mice with or without W_csb_ administration and in controls. Occludin positivity was lower in MCD mice compared to the CTR but was higher after W_csb_ administration when compared to untreated MCD mice. Original magnification: 20×. In (**a**,**b**), histograms show means and standard deviations for villi length and occludin positivity score. Data from each animal are plotted as solid circles in the graph. * *p* < 0.05 vs. all groups; ° *p* < 0.05 vs. CTR.

LPS translocation induced by an MCD diet could be related to injury of the ileal mucosal barrier integrity. Therefore, we evaluated the terminal ileum of mice in our experimental model (Figure 5). For this set of experiments, we focused on mice treated for 8 weeks, given the higher degree of liver damage at this time point. H&E and PAS staining were used to observe the intestinal structure.

In MCD mice, the intestinal villi were shortened indicating structural damage induced by the diet; accordingly, the length of intestinal villi in MCD-8wk mice was lower (100.2 ± 39.8 µm) compared to control mice (155.5 ± 39.0 µm; *p* < 0.001).

Next, the positivity for intestinal occludin, a tight junction protein, was determined through immunofluorescence to evaluate the intestinal mucosal barrier integrity. The occludin positivity in enterocytes was significantly lower in MCD mice (score = 1.6 ± 0.5) compared to controls (score = 3.6 ± 0.5; *p* < 0.001); MCD/W_csb_ mice showed a significantly higher enterocyte occludin positivity (score = 2.6 ± 0.5) compared to MCD mice (*p* < 0.05).

## 3. Discussion

In the present study, we demonstrated that, in MCD mice, treatment with calcium-sulphate-bicarbonate water was associated with: (i) liver histopathology improvement, lower inflammation, fibrosis, collagen deposition, and a lower activation rate of fibrogenetic cells; (ii) lower hepatocyte localization of LPS; (iii) reduced TLR4 pathway activation, with less TLR4+ macrophages and nuclear pNF-κB positivity in hepatocytes; iv) a preserved terminal ileum histopathology, with longer villi and higher occludin positivity in enterocytes.

The term hydropinotherapy defines the act of drinking natural mineral waters, especially thermal waters, for therapeutic purposes [20]. This approach represents a useful tool in support of pharmacological therapies, especially in inflammatory diseases. Natural mineral waters, compared to tap water, are characterized by a high concentration of minerals and elements, such as calcium, carbonic metabolites, sodium chlorite, sulphates, and/or iron, in varying proportions according to the source [16]. Based on the composition, particularly with regard to the prevalent elements, different waters may have distinct beneficial effects on health. Interestingly, mineral waters have been empirically known for their beneficial effects on liver and biliary disorders. Recently, scientific evidence for the positive effects of mineral water compounds has been emerging, supporting their use for clinical purposes. The use of mineral water in patients affected by biliary disorders has been reported to modulate the severity of symptoms due to the altered bile flow [21] and was also associated with a reduced lithogenic risk and metabolic improvements from the regulation of bile composition [17]. Furthermore, mineral-rich waters can exert positive effects on fat and carbohydrate metabolisms in a murine model of metabolic syndrome [22].

In addition to the liver, mineral water administration was shown to have beneficial effects on the intestinal mucosa and gut microbiota, both in human and animal models. Bicarbonates contribute to intestinal barrier constitution, and administration of bicarbonate-rich water has been shown to have positive effects on intestinal histopathology [23,24]. Interestingly, sulphate water administration can modify gut microbiota, by favoring hydrogen sulfide production through sulphate-reducing bacteria (such as *E. Coli*) in the intestinal lumen, exerting local anti-inflammatory effects [23,25,26].

Indeed, a bidirectional relationship between the gut and the liver has been identified both in health and disease [8]; the so-called gut–liver axis is characterized by the direct afflux of nutrients and other substances from the intestine to the liver via the portal vein. However, pathological conditions affecting the gastrointestinal tract can cause a disruption to intestinal barrier integrity, thereby resulting in the translocation of potentially harmful compounds to the liver [8]. An emerging role of the gut–liver axis modification in the pathogenesis of NASH has been underlined. In particular, NASH is associated with an increased intestinal permeability, which is largely due to the alterations in gut microbiota composition, both in human subjects and in animal models. In this context, the altered nutritional regimen has a direct effect in determining the bacterial populations in the gut, favoring the eventual translocation of PAMPs to the liver [8]. Among PAMPs, LPS translocation to the liver can lead to the recruitment and activation of proinflammatory cells contributing to disease progression in human and experimental NASH [11].

In this scenario, in a murine model of NASH, we tested whether a natural mineral water (rich in calcium, sulphates, and bicarbonate) could have potential beneficial effects on liver histopathology through the modulation of liver LPS localization derived from gut translocation. Our investigation showed that mice with NASH, treated with W_csb,_ were characterized by a slight but significant improvement in liver inflammation and fibrosis but not of hepatocyte steatosis compared to the untreated ones. In accordance with this, the increase in αSMA+ activated stellate cells was reduced in W_csb_ treated mice. Furthermore, we reported LPS localization within hepatocytes in NASH mice, which was reduced in mice treated with mineral water administration. In a previous study [10], liver LPS localization was associated with the activation of TLR4/NF-κB pathways. TLR4 is a pattern recognition receptor (PRR) which can bind both to DAMPs and PAMPs; it is implied in the immune response to endotoxins from the activation of the transcriptional factor NF-κB both in macrophages and in hepatocytes [27]. Interestingly, the administration of W_csb_ to NASH mice was associated with a lower number of TLR4+ macrophages within the liver and a reduction of pNF-κB in hepatocytes. Taken together with the reduced lobular inflammation, these data suggest that natural mineral water could potentially exert a modulatory effect on bacterial translocation and TLR4 pathway activation, leading to the observed improvements to liver injury.

To further support this hypothesis, we examined the histopathology of the terminal ileum and the integrity of the intestinal barrier. Bacterial translocation and endotoxemia in NASH are associated with an impairment of intestinal barrier integrity [28] and increased intestinal permeability [8]. In the MCD mouse model, intestinal dysbiosis was described together with alterations in intestinal mucosa [29,30]. Accordingly, we observed structural alterations of the intestinal mucosa of MCD mice, characterized by shorter villi and reduced occludin positivity on enteric epithelium compared to controls. Remarkably, in MCD mice treated with W_csb_, histological injury in the terminal ileum was less prominent compared to MCD mice alone; moreover, occludin localization between enterocytes was improved after mineral water treatment, suggesting that the positive effects of mineral water in these mice are also present on the terminal ileum and barrier integrity. Interestingly, it has been shown that bicarbonate-sulphate-calcium-magnesium water exerts positive effects on indirect markers of gut–liver axis activation and in modifying gut microbiota in patients with NAFLD in a prospective longitudinal interventional study [31].

### 3.1. Limitations of the Study

Our study represents a preliminary report of the positive effects of calcium-sulphate-bicarbonate water in NASH mice and the gut–liver axis.

- NASH model. The MCD model does not replicate the NAFLD-related metabolic syndrome. In this model, the administration of a methionine-choline-deficient diet determines the development of hepatic steatosis, together with prominent inflammation and fibrosis; this is due to an impaired VLDL metabolism caused by methionine and choline deficiency. In turn, this leads to lipid accumulation in hepatocytes and subsequent lipotoxic injury, then progression towards NASH [32]. In the present study, we chose MCD instead of other models because it replicates the inflammatory and fibrogenetic injuries occurring in NASH more consistently than other models, thereby allowing us to better study the potential inflammatory response to LPS in the liver [29,30]. On the other hand, the model is not suitable for evaluating the potential beneficial effects of mineral water on glucose and lipid metabolisms [19,33]; accordingly, we did not observe significant effects on hepatic steatosis, and future studies are necessary to expand the investigations on metabolic features by using other disease models.
- Mechanistic study. Here, we reported an association between thermal water administration, liver and intestine histologic improvement, as well as LPS hepatocyte localization. However, future analyses should be aimed at performing an in-depth assessment of the mechanisms responsible for the modulation of inflammatory and fibrogenetic injuries of the liver, in addition to confirming the modulatory properties on the intestinal barrier and microbiota [18,34].

### 3.2. Conclusions and Future Perspectives

**Figure 6:**
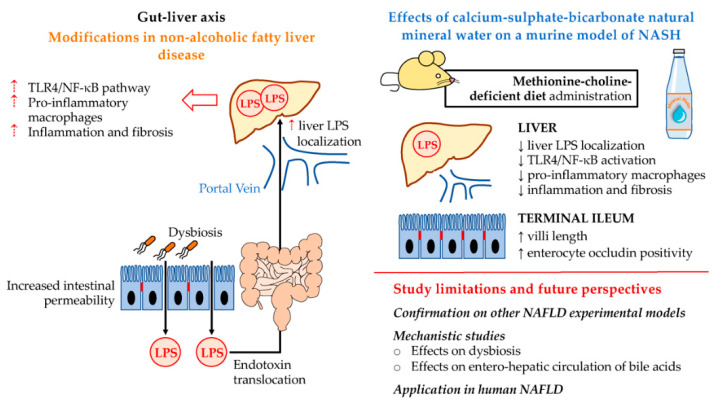
Framework figure. The gut–liver axis represents a key mechanism in nonalcoholic fatty liver disease (NAFLD) progression towards nonalcoholic steatohepatitis (NASH), triggering the activation of toll-like receptor (TLR) 4 and nuclear factor-k B (NF-kB) pathways in the liver via translocation of endotoxins (e.g., lipopolysaccharide: LPS). This is due to altered intestinal permeability and gut microbiota composition. In the present study, we investigated the effects of calcium-sulphate-bicarbonate natural mineral water on the methionine-choline-deficient diet model of NASH. Mineral water administration was associated with improvements in liver and terminal ileum histopathology. Further studies should be performed in order to confirm the findings of other NAFLD models; clarify the mechanisms at the basis of these beneficial effects; and test the eventual possible application of mineral water administration to patients as a supporting therapy for NAFLD/NASH.

In conclusion, our study describes the effects of natural mineral water rich in calcium, sulphates, and bicarbonates on liver and terminal ileum histopathology, suggesting a possible modulation of the gut–liver axis and of inflammatory injury as mediated by LPS and TLR4 pathways (Figure 6).

Future studies in humans may allow us to evaluate whether the administration of W_csb_ could represent a helpful addition to diet and physical activity in NAFLD/NASH or MAFLD subjects. Particularly, the absorption of nutrients from water can be affected by food; therefore, future studies translating these findings in a human setting should take into account dietary regimen and diet composition [14,15]. Moreover, clinical research on therapeutic applications of calcium-sulphate-bicarbonate water in NAFLD should include a detailed assessment of patient behavior, lifestyle intervention and physical activity, in order to obtain reproducible results and definitive evidence of mineral water efficacy [18,35]. Finally, future mechanistic studies in experimental models should be focused on the effects of natural mineral waters on the entero-hepatic circulation of bile acids; this is a pivotal mechanism at the basis of the gut–liver axis that influences NASH development [8].

## 4. Materials and Methods

### 4.1. Experimental Setting

Eight-week-old male C57BL/6 mice were purchased from Charles River Laboratories (Charles River UK Ltd., Margate, UK). Mice were divided into the following experimental groups:

- Controls: CTR (N = 10)
- MCD+Tap water: MCD (N = 10)
- MCD+Calcium-sulphate-bicarbonate water: MCD/W_csb_ (N = 10).

Mice were housed in a dedicated facility at Sapienza University of Rome in compliance with Italian regulations. Mice were kept in a room maintained at a temperature of 23 ± 1 °C and 50 ± 10% relative humidity, with food and water available ad libitum. The animal room was on a 12:12 h light:dark cycle. Mice were individually identified by ear punching.

Control mice were fed with a normal chow diet and were randomly divided into mice drinking tap or calcium-sulphate-bicarbonate water at room temperature. No differences were revealed in terms of weight, serum analysis, or histopathology; therefore, these mice were grouped and considered altogether as controls.

Tap water from the Rome aqueduct is microbiologically safe (https://www.gruppo.acea.it/al-servizio-delle-persone/acqua/acea-ato-2/la-qualita-della-tua-acqua accessed on 16 August 2022) and natively contains low levels of bicarbonates (395 mg/L), calcium (104 mg/L), and sulphates (16.6 mg/L). For mice consumption, tap water was filtered through a water softener system and treated with a 0.25% solution of sodium hypochlorite to further avoid microbial contamination. Bottled natural mineral water was obtained from Terme di Chianciano SpA (http://acqueminerali.it accessed on 16 August 2022) and contained high concentrations of calcium (715 mg/L), sulphates (1840 mg/L), and bicarbonate (842 mg/L).

Steatohepatitis was induced in mice through a methionine-choline deficient (MCD) diet as previously reported. The amount of water consumed by the animals was measured daily. Drinking bottles were replaced once per day. Weight was measured weekly.

Mice were euthanised after 4 weeks (N = 5 per group) or 8 weeks (N = 5 per group) of treatment. At the time of euthanasia, mice were anaesthetized using saturated diethyl ether vapor and euthanized by cervical dislocation. Blood samples were drawn from a cardiac puncture. The liver and terminal ileum were harvested and processed for histology.

The study protocol (n° 553/2019-PR) was approved by the Italian Ministry of Health and by the university commission for animal care following the criteria of the Italian National Research Council. Animals received humane care according to the criteria outlined in the “Guide for the Care and Use of Laboratory Animals” prepared by the National Academy of Sciences and published by the National Institutes of Health.

### 4.2. Histopathology, Immunohistochemistry and Immunofluorescence

Tissue samples were fixed in formalin and embedded in paraffin. Three-micron sections were obtained, and routine histological stains were performed, including hematoxylin-eosin (H&E) and Sirius Red/Fast Green (SR/FG).

For immunohistochemistry, endogenous peroxidase activity was blocked by a 30min incubation in methanolic hydrogen peroxide (2.5%). Antigens were retrieved, as indicated by the vendor, by applying Proteinase K (Dako, Glostrup, Denmark, code S3020) for 10 min at room temperature. Sections were then incubated overnight at 4 °C with primary antibodies (Table 1). All primary antibodies are of commercial use; have been tested and validated by manufacturers; and have been previously used and published [11]. For all immunoreactions, negative controls (the primary antibody was replaced with preimmune serum) were also included [36,37].

Then, samples were rinsed twice with phosphate buffered saline (PBS) for 5 min, incubated for 20 min at room temperature with a secondary biotinylated antibody, and then with Streptavidin-horseradish peroxidase (LSAB+, Dako, Glostrup, Denmark code K0690). Diaminobenzidine (Dako, Glostrup, Denmark code K3468) was used as a substrate, and sections were counterstained with hematoxylin.

For immunofluorescence, nonspecific protein binding was blocked using specimen incubation with 5% normal goat serum and then with primary antibodies overnight. Sections were incubated for 1 h at room temperature with labelled isotype-specific secondary antibodies (AlexaFluor^®^, Invitrogen, Life Technologies Ltd., Paisley, UK) and counterstained with 4,6-diamidino-2-phenylindole (DAPI) for visualization of cell nuclei [36,37].

Sections were examined in a coded fashion with a Leica Microsystems DM 4500 B Light and Fluorescence Microscopy (Weltzlar, Germany), equipped with a video-camera (Jenoptik Prog Res C10 Plus Videocam, Jena, Germany) by two independent researchers. Slides were further scanned with a digital scanner (Aperio ScanScope^®^ CS and FL Systems; Aperio Digital Pathology, Leica Biosystems, Milan, Italy) and processed using ImageScope.

Histopathological evaluation of the liver was performed according to previous indications and included scoring of hepatocyte steatosis, hepatocyte hypertrophy, lobular inflammation, and fibrosis [43]. Moreover, on SR/FG-stained slides, collagen deposition was automatically calculated with an algorithm and expressed as a positive area percentage [11]. Terminal ileum samples were radially cut; mean villus length was quantified from ten nonoverlapping 20× fields per mouse on H&E-stained slides [44,45].

The number of α-smooth muscle actin (αSMA)+ hepatic stellate cells (HSCs) and portal/periportal myofibroblast, and the number of F4/80+ or TLR4+ macrophages were calculated as the number of cells per High Powered Field (HPF, i.e., 40× magnification). For each slide, at least 10 nonoverlapping microscopic HPFs were randomly chosen [46].

For LPS positivity, the percentage of positive hepatocytes was automatically calculated by an algorithm for the entire section. The nuclear localization of pNF-κB by hepatocytes was automatically calculated using specific algorithms for the entire section [11].

The occludin positivity in the ileum was measured by counting the percentage of positive epithelial cells with respect to the total number of epithelial cells and expressed as a semiquantitative score (0 ≤ 1%; 1 = 1–10%; 2 = 10–30%; 3 = 30–50%; 4 ≥ 50%).

### 4.3. Statistical Analysis

Continuous variables were expressed as mean ± standard deviation. Mann–Whitney U-test was used to study differences among groups. All tests were two-tailed, and a statistical significance was set at a *p*-value of less than 0.05. Analyses were performed using computer software packages (IBM SPSS Statistics v20.0, Armonk, NY, USA).

## Figures and Tables

**Table 1:** List of primary antibodies.

Antibody	Host	Manufacturer	Code	Dilution	Application	Reference
αSMA	Rabbit	abcam	ab150301	1:100	IHC/IF	[38]
*E. coli* LPS	Mouse	abcam	ab35654	1:50	IHC/IF	[39,40]
Occludin	Rabbit	abcam	ab216327	1:100	IF	[41]
pNF-κB	Rabbit	SCBT	sc-33039	1:100	IHC/IF	[42]
TLR4	Rabbit	Atlas Antibodies	HPA049174	1:200	IHC/IF	[11]

List of abbreviations. αSMA: α-smooth muscle actin; IF: immunofluorescence; IHC: immunohistochemistry; LPS: lipopolysaccharides; pNF-κB: phosphorylated nuclear factor-κB; TLR: toll-like receptor. Manufacturers. abcam, Cambridge, UK; Atlas Antibodies, Bromma, Sweden; SCBT: Santa Cruz Biotechnology Inc., Dallas, TX, USA.

## Data Availability

Data is available from the Authors upon reasonable request.
